# Ten-Hour Exposure to Low-Dose Ketamine Enhances Corticostriatal Cross-Frequency Coupling and Hippocampal Broad-Band Gamma Oscillations

**DOI:** 10.3389/fncir.2018.00061

**Published:** 2018-08-13

**Authors:** Tony Ye, Mitchell J. Bartlett, Matthew B. Schmit, Scott J. Sherman, Torsten Falk, Stephen L. Cowen

**Affiliations:** ^1^Department of Psychology, University of Arizona, Tucson, AZ, United States; ^2^Department of Pharmacology, University of Arizona College of Medicine, Tucson, AZ, United States; ^3^Department of Neurology, University of Arizona College of Medicine, Tucson, AZ, United States; ^4^Graduate Interdisciplinary Program in Neuroscience, University of Arizona, Tucson, AZ, United States

**Keywords:** cross-frequency coupling, depression, schizophrenia, Parkinson’s disease, gamma, high-frequency oscillation, levodopa-induced dyskinesia

## Abstract

**Introduction:** Treatment-resistant depression, post-traumatic stress disorder, chronic pain, and L-DOPA-induced dyskinesia in Parkinson’s disease are characterized by hypersynchronous neural oscillations. Sub-anesthetic ketamine is effective at treating these conditions, and this may relate to ketamine’s capacity to reorganize oscillatory activity throughout the brain. For example, a single ketamine injection increases gamma (∼40 Hz) and high-frequency oscillations (HFOs, 120–160 Hz) in the cortex, hippocampus, and striatum. While the effects of single injections have been investigated, clinical ketamine treatments can involve 5-h up to 3-day sub-anesthetic infusions. Little is known about the effects of such prolonged exposure on neural synchrony. We hypothesized that hours-long exposure entrains circuits that generate HFOs so that HFOs become sustained after ketamine’s direct effects on receptors subside.

**Methods:** Local-field recordings were acquired from motor cortex (M1), striatum, and hippocampus of behaving rats (*n* = 8), and neural responses were measured while rats received 5 ketamine injections (20 mg/kg, i.p., every 2 h, 10-h exposure). In a second experiment, the same animals received injections of D1-receptor antagonist (SCH-23390, 1 mg/kg, i.p.) prior to ketamine injection to determine if D1 receptors were involved in producing HFOs.

**Results:** Although HFOs remained stable throughout extended ketamine exposure, broad-band high-frequency activity (40–140 Hz) in the hippocampus and delta-HFO cross-frequency coupling (CFC) in dorsal striatum increased with the duration of exposure. Furthermore, while ketamine-triggered HFOs were not affected by D1 receptor blockade, ketamine-associated gamma in motor cortex was suppressed, suggesting involvement of D1 receptors in ketamine-mediated gamma activity in motor cortex.

**Conclusion:** Prolonged ketamine exposure does not enhance HFOs in corticostriatal circuits, but, instead, enhances coordination between low and high frequencies in the striatum and reduces synchrony in the hippocampus. Increased striatal CFC may facilitate spike-timing dependent plasticity, resulting in lasting changes in motor activity. In contrast, the observed wide-band high-frequency “noise” in the hippocampus suggests that ketamine disrupts action-potential timing and reorganizes connectivity in this region. Differential restructuring of corticostriatal and limbic circuits may contribute to ketamine’s clinical benefits.

## Introduction

Ketamine is a widely available FDA-approved drug that was developed in the 1960s as an anesthetic (Domino et al., 1965). In the last decade, the potential therapeutic applications of ketamine have expanded considerably. For example, hour to days-long exposure to sub-anesthetic ketamine can provide weeks-to-month management of treatment-resistant depression (Berman et al., 2000; Diamond et al., 2014; Andrade, 2017), post-traumatic stress disorder (PTSD) (Feder et al., 2014), chronic pain (Niesters et al., 2014), and L-DOPA-induced dyskinesias (LID) (Bartlett et al., 2016; Sherman et al., 2016). Although the mechanisms underlying ketamine’s clinical effectiveness are not well understood, progress has been made toward understanding the action of ketamine and its metabolites upon *N*-Methyl-D-aspartate (NMDA), α-amino-3-hydroxy-5-methyl-4-isoxazolepropionic acid (AMPA), opioid, and dopamine receptors (Sleigh et al., 2014; Zanos et al., 2016), and ketamine’s capacity to trigger profound changes in oscillatory activity throughout the brain (Hunt et al., 2006; Lazarewicz et al., 2010; Nicolás et al., 2011; Caixeta et al., 2013; Muthukumaraswamy et al., 2015; Shaw et al., 2015).

The optimal duration of ketamine exposure for the treatment of neurological diseases has not been established, and the effects of extended exposure on neural activity is not understood. In the clinic, exposure duration can vary considerably. For example, while the majority of initial human studies investigating the anti-depressant effects of ketamine used a single 40-min infusion, later publications report improved outcomes using 3–6 repeated 40–min infusions over days to weeks (Murrough et al., 2013; Shiroma et al., 2014; Singh et al., 2016; Vande Voort et al., 2016), or longer 100-min exposure sessions (Rasmussen et al., 2013). Far longer exposures are used for treating chronic pain where continuous ketamine exposure can last 72–96 h (Noppers et al., 2010). Our original inspiration for the present study was preclinical (Bartlett et al., 2016) and clinical case-study data (Sherman et al., 2016) that showed that extended ketamine exposure results in weeks-long reductions in LID. In these cases, exposure was 72–96 h in humans (Sherman et al., 2016) and 10-h in rats (Bartlett et al., 2016). These infusion times were based on ketamine protocols used to treat pain states, such as intractable migraines (Niesters et al., 2014), where extended exposure durations can reduce pain for 2–3 months (Sigtermans et al., 2009). It is conceivable that extended ketamine exposure could also improve the treatment of treatment-resistant depression as the typical 40-min infusions for depression improve symptoms for only ∼2 weeks on average. Longer exposure times may improve outcomes (Rasmussen et al., 2013). Recently, a small feasibility clinical trial using 96-h ketamine infusions to treat treatment-resistant depression supports the tolerability of extended exposure in this patient population (Lenze et al., 2016).

Almost all investigations of the neural responses to ketamine exposure have investigated the effects following a single injection. Thus, little is known about the effects of longer exposure durations on neural activity and neural systems. A second outstanding question is how extended clinical exposure to ketamine during a 5- to 10-h infusion session provides long-term relief, despite ketamine itself being metabolized within hours (Páleníček et al., 2011). It is therefore conceivable that extended periods of exposure further enhance ketamine’s capacity to reorganize neural circuitry (Duman et al., 2012) and alter glutamatergic signaling (Li et al., 2016). This is suggested by a number of observations. For example, low-dose ketamine exposure enhances synchrony between neurons in multiple brain regions at low (1–7 Hz) and high (∼140 Hz) frequencies (reviewed in Hunt and Kasicki, 2013). Such synchronization at low doses may support spike-timing dependent plasticity (Bi and Poo, 1998; Masquelier et al., 2009), which may contribute to ketamine’s capacity to alter synaptic morphology (Phoumthipphavong et al., 2016). Thus, identifying changes in neural activity that emerge during extended ketamine exposure could identify circuit-level mechanisms involved in ketamine’s therapeutic effectiveness at low doses.

Sub-anesthetic ketamine exerts diverse effects on neural synchrony (Hunt and Kasicki, 2013). For example, a single low-dose injection triggers a rapid increase in gamma (40–80 Hz) and high-frequency oscillations (HFOs, 120–160 Hz) in the striatum (Olszewski et al., 2013), cortex (Hakami et al., 2009; Nicolás et al., 2011; Shaw et al., 2015), and hippocampus (Caixeta et al., 2013). Low-dose ketamine also enhances cross-frequency coupling (CFC) which measures the degree to which the timing of high-frequency oscillations are organized by the phase of low-frequency oscillations. Increased CFC may support neural plasticity by organizing the precise timing of action potentials (Canolty and Knight, 2010; Lisman and Jensen, 2013). Injection of sub-anesthetic doses of ketamine increases theta (5–10 Hz)-gamma and theta-HFO CFC in the hippocampus (Caixeta et al., 2013) and delta (1–4 Hz)-HFO CFC in the cortex and striatum (Cordon et al., 2015). Some of these effects are likely due to ketamine’s antagonism of NMDARs as injection of the selective non-competitive NMDAR antagonist MK-801 also triggers HFOs and gamma in the cortex (Pinault, 2008; Hakami et al., 2009) and HFOs in the ventral striatum (Hunt et al., 2006; Olszewski et al., 2013).

Ketamine induces strong oscillatory activity and produces behavioral and therapeutic effects that last long after ketamine-induced NMDAR blockade subsides. Consequently, we hypothesized that 10-h exposure to ketamine, as opposed to a single acute exposure, will entrain activity within corticostriatal circuits such that ketamine-induced oscillations become progressively enhanced over the course of exposure. To test this, we measured oscillatory activity during a 10-h exposure to low-dose ketamine using the rodent prolonged infusion protocol described in Bartlett et al. (2016), and recorded local-field potentials (LFPs) from motor cortex (M1), ventral striatum (vSTR), dorsal striatum (dSTR), and hippocampus (HC) of awake and behaving rats. Corticostriatal regions (dSTR and M1) were chosen given their involvement in motor pathophysiology (Dupre et al., 2016), and vSTR and HC were selected given their involvement in depression and PTSD (Sailer et al., 2008; Dunkley et al., 2014). Our data suggest that sub-anesthetic ketamine’s lasting effect on neural synchrony may not reside in its capacity to produce sustained HFOs, but, instead, in its capacity to increase CFC in corticostriatal circuits and to trigger broad-band high-frequency (>30 Hz) neural activity in the hippocampus.

## Materials and Methods

### Animals and Surgical Procedures

**FIGURE 1:**
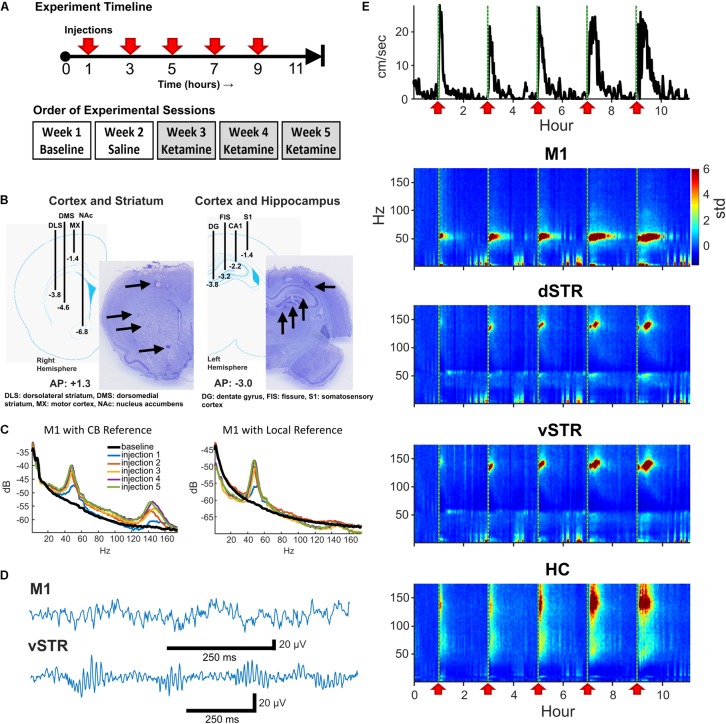
Experiment design and neural recordings. **(A)** Top: Timeline of an individual experimental session. Five sub-anesthetic doses of ketamine (20 mg/kg, i.p.) or saline were injected throughout each 11+ hour recording session. One hour of pre-injection baseline was recorded prior to the first injection. Bottom: Timeline for experimental sessions. Each animal received a single ketamine or saline injection recording session each week. **(B)** Schematic of electrode array placement in the right-hemisphere (AP: +1.3, ML: +2.7 centered, DV: -6.8 deepest electrode) and the left-hemisphere (AP: -3.0, ML: -2.2 centered, DV: -3.8 deepest electrode) and exemplar histological sections. **(C)** Local-field electrodes were referenced to within-region electrodes to reduce contamination from volume conduction. To illustrate the effects of re-referencing, the power-spectral density (PSD) from an M1 electrode referenced to the cerebellar ground screw (left) or to a second within-region (M1) electrode (right) are presented. Recordings were acquired 10-min prior to ketamine injection (black) and 15 min following each ketamine injection (colored traces). Note the reduction in HFO power when using a local reference indicating that HFO activity was volume conducted. **(D)** Exemplar local-field traces of ketamine-induced gamma (M1) and HFOs (vSTR) recorded 15 min after injection. **(E)** Animal movement (top trace) and power spectrograms acquired from electrodes in M1, dSTR, vSTR, and HC. Ketamine injection times are indicated as vertical dashed lines and red arrows. Values are in standard deviations above the mean measured during the 30 min period preceding the first injection.

Eight male Sprague-Dawley rats (∼350 g at surgery, Envigo, Indianapolis, IN, United States) were housed individually in a 12-h reverse light/dark cycle (i.e., lights off from 9AM to 9PM) room with food and water available *ad libitum*. Rats were anesthetized with isoflurane and implanted with two electrode arrays, each array composed of 16 stereotrodes (California Fine Wire Co., Grover Beach, CA, United States). A skull screw over cerebellum was reference and ground. The first electrode array was centered at AP: +1.3, ML: +2.7, right hemisphere, with stereotrodes targeting M1 (DV: -1.4), dorsolateral striatum (DLS, DV: -3.8), dorsomedial striatum (DMS, DV: -4.6), and nucleus accumbens (NAc, DV: -6.8, **Figure 1B**). The second array targeted left HC and cortex (centered at AP: -3.0, ML: +2.2) with electrodes lowered near the fissure (DV: -3.2), CA1 (DV: -2.3), dentate gyrus (DV: -3.8), and S1 (DV: -1.4). Rats recovered for 1 week post-surgery. Animals were provided with post-operative analgesia. Carprofen (Zoetis, Parsippany, NJ, United States) was delivered (5 mg/kg, *s.c.*) for 48 h following surgery. Topical anti-biotic ointment (Water-Jel Technologies, Carlstadt, NJ, United States) was applied to the incisional site for up to 5 days as needed. Sulfamethoxazol and Trimethoprim (Hi-tech Pharmacal Co., Inc., Amityville, NY, United States) was administered orally (15 mg/kg) daily until conclusion of experiments.

Procedures were in accordance with NIH guidelines for the Care and Use of Laboratory Animals and approved IACUC protocols at the University of Arizona. One animal was eliminated from the study due to excessive recording artifacts.

### Drug Treatments

On a given 11-h neural recording session for Experiment 1, animals received a total of five injections of ketamine (20 mg/kg, i.p.) or saline. Injections were administered every 2 h as described in Bartlett et al. (2016) and as summarized in **Figure 1A** (top). This paradigm is identical to that used by Bartlett et al. (2016) to model clinical infusions in rodents, and it was chosen as it has been shown to reduce levodopa-induced dyskinesia (LID). Multiple injections within a session ensured that serum concentrations of ketamine remained high throughout exposure. The 2-h spacing between injections was important given the faster metabolism of ketamine in rodents (∼ 1 h, Páleníček et al., 2011; Veilleux-Lemieux et al., 2013) relative to humans (∼3 h, Clements et al., 1982). Recording sessions involved injections of either ketamine hydrochloride (20 mg/kg, Clipper Distributing, St. Joseph, MO, United States) or 0.9% saline according to the schedule illustrated on the top of **Figure 1A**. Each animal underwent one 11-h baseline session, one saline injection session, and three successive ketamine sessions over the course of 5 weeks (**Figure 1A**, bottom). Multiple ketamine sessions for each animal were incorporated into the experiment to investigate whether repeated exposure over weeks altered neural responses (see **Figure 4**). Although every animal underwent 3 ketamine exposure sessions, data from 5 of the 24 ketamine exposure sessions were eliminated from the analysis of physiological responses due to poor recording quality (1 or 2 sessions were dropped from 3 of the 8 rats).

One week after the completion of Experiment 1, the same animals were used for a second experiment (Experiment 2) in which the involvement of dopamine D1 receptors in ketamine-induced oscillatory activity was investigated. In this experiment rats received injections of a D1-antagonist (SCH-23390, Tocris Bioscience, Minneapolis, MN, United States) and ketamine (20 mg/kg). Each session began with a 1-hr baseline. A single injection of SCH-23390 (1 mg/kg, i.p.) was administered followed by a single injection of ketamine 15 min after the injection. This procedure was repeated again after 2 h. After another 2 h rats received a single injection of SCH-23390.

### Neurophysiological Recordings

Data was acquired from a multi-channel data acquisition system (KJE-1001, Amplipex Ltd.). The digitizing headstage was connected to the animal, and the signal was sent to the recording system through a lightweight tether and commutator. A light-emitting diode (LED) was attached to the rat’s implant for video tracking (Camera: Manta G-033C, Allied Vision, Exton, PA, United States). Recording sessions were conducted once per week for each animal (as in **Figure 1A**) with experiments starting at 5AM. The animals and their home cage were transported to the recording room, and remained in their home cage during recordings sessions with food and water available *ad libitum*.

### Histology

At the conclusion of the experiment, rats were deeply anesthetized and electrolytic lesions were produced at recording sites via direct current stimulation (20 mA, 20 s). Rats were sacrificed 3 days later with Euthasol (0.35 mg/kg, i.p.; Virbac, Fort Worth, TX, United States) and brains were extracted through transcardial perfusion with phosphate buffered saline (PBS) followed by 4% paraformaldehyde in PBS. Prepared 40-μM thick coronal sections were cut using a frozen microtome and Nissl stained for verification of electrode placement (**Figure 1B**).

### Data Analysis

Analysis was performed via custom-written code [MATLAB: MathWorks Inc., Natick, MA, United States and R: R Core Team (2017)]. Local-field signals were acquired at 20,000 Hz and down sampled to 500 Hz. Local-field traces were analyzed for artifacts through visual inspection and automatic artifact detection. Artifacts can come from multiple sources such as cable artifact and electromyographic (EMG) signal. The 2-min periods before and after each injection were excluded from analysis to avoid potential artifacts created by the experimenter entering the room and picking up and injecting the animal. Candidate artifacts in the LFP signal were identified as periods where the absolute value of local-field traces exceeded 1.5 mV or when summed cross-band power (2–160 Hz) exceeded the 99.98th percentile.

### References and Addressing Volume Conduction

Interpreting LFP signals as being local to a target region is complicated by contamination from volume-conducted signals from other regions or from EMG artifacts. One approach to address this is to reference signals from one electrode to a second within-region electrode. The capacity of rereferencing to localize region-specific and ketamine-induced oscillations has been previously reported (Hunt et al., 2011). Thus, to be confident that the measured signal was local, we followed the following procedure: **M1:** The M1 electrode was referenced to a second M1 electrode 0.7 mm away and at the same depth (1.4 mm). **HC:** To ensure the signal was local to the HC and integrated the diverse oscillatory activity in the HC, the signal from the HC fissure electrode was referenced to the CA1 electrode (1-mm distance). **Striatum:** The geometry of the array allowed segregation of dorsal and ventral striatum, but the array did not have an arrangement allowing segregation of responses within subregions such as the nucleus accumbens core/shell. Consequently, we conservatively divided striatal recordings into dorsal and ventral components. This was accomplished by referencing the dorsolateral striatum electrode against the dorsomedial electrode to yield a dSTR signal, and referencing the nucleus accumbens (NAc) electrode against the dorsomedial electrode to yield a vSTR signal (see **Figure 1B**). **Figure 1C** presents the power-spectral density measured before (cerebellar reference) and after (local M1 reference) re-referencing, suggesting that HFOs in M1 are volume conducted. The effects of re-referencing on HFOs varied by region, and an example of the consequences of re-referencing on power-spectral responses in all regions is presented in **Supplementary Figure 1**.

### Spectral Power

Spectral power across frequency bands was determined using a fast Fourier transform spectrogram [frequency bin = 0.5 Hz, 10-s Hanning window, spectrogram() in Matlab]. A complex wavelet convolution spectrogram [Morlet family, cycles = 5, cwt() in Matlab] was only used to visualize spectral power for the analysis of cross-frequency coupling (see next section) given the high temporal resolution of the wavelet convolution. Statistical analysis of oscillatory power was restricted to the following frequency bands: delta (1–4 Hz), theta (5–10 Hz), beta (15–30 Hz), gamma (35–55 Hz), and HFO (120–160 Hz). Normalization: To address the issue of power-law 1/f scaling, data was normalized using the Z-transform method as described in Cohen (2014). This was accomplished by first subtracting the baseline mean from the spectral power and then dividing this value by the standard deviation to yield a *z* score. The baseline period was defined as the -32 to -2 min interval preceding the first injection.

### Cross-Frequency Coupling

Phase-amplitude cross-frequency coupling (PAC) was measured as described in Cohen (2014). First, LFP signals were filtered in the target low- and high-frequency bands using a Butterworth infinite impulse response (IIR) filter (fs = 500 Hz, order = 6). Phase was extracted using a Hilbert transform, and power was extracted as the envelope of the absolute value of the filtered signal. CFC was computed as $PAC=|n^{-1}\Sigma_{t=0}^{n}a_{t}e^{{i\varphi }_{t}}|$ where *a* is high-frequency power and φ is the phase of the low-frequency signal. This value was compared to values computed using a randomized shuffle control where the original low-frequency signal was randomly shifted in time on each permutation (*n* = 200 permutations). The mean and standard deviation of this null hypothesis distribution were used to convert the measured PAC score into a *z* score (PACz).

### Statistical Analyses

Statistical significance was assessed using ANOVA or Student’s *t*-test (α = 0.05). Tukey’s honest significance test or Holm–Bonferroni method were used to adjust *p*-values for *post hoc* comparisons unless otherwise stated. The Shapiro–Wilk test of normality [shapiro.test() in R] was used to determine if the data was normally distributed, and the data used for ANOVA satisfied this criterion (Shapiro–Wilk *p* > 0.05).

## Results

### A Single Injection of Sub-anesthetic Ketamine Triggers Gamma Oscillations in M1, HFOs in Striatum, and Broadband Activity in Hippocampus

**FIGURE 2:**
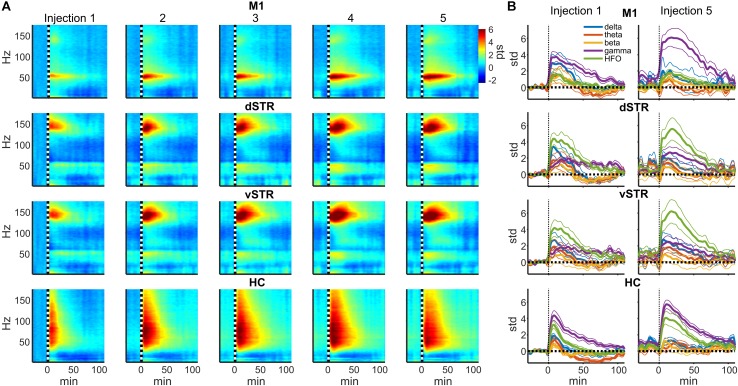
Ketamine-induced oscillatory activity. **(A)** Spectral responses aligned to the time of each of the 5 successive ketamine injections. Spectra for each recording session were averaged (19 sessions from *n* = 8 rats). Each row indicates the response for each brain region and each column indicates the injection number. Units are in standard deviations above or below the mean spectral power measured during the –32 to –2 min period preceding the first injection. Spectral power was smoothed in time with a 5-min Hanning window prior to averaging. **(B)** Time course of spectral responses following ketamine injections 1 and 5 by frequency band. Lines indicate mean ± SEM (*n* = 8 rats).

In rodents and humans, administration of sub-anesthetic ketamine triggers robust high-frequency activity throughout the brain (Nicolás et al., 2011; Caixeta et al., 2013; Cordon et al., 2015; Muthukumaraswamy et al., 2015). Our data support these observations as ketamine injections (20 mg/kg) induced powerful and region-specific gamma oscillations and HFOs. Exemplar local-field traces for M1 and vSTR are presented in **Figure 1D**. Spectrograms recorded from all 4 regions during a single experimental session are presented in **Figure 1E**. The mean spectral responses surrounding each ketamine injection are shown in **Figures 2A,B**. Mean spectral responses were computed by first determining an average response for each of the 8 rats. Each rat received multiple ketamine exposure sessions (**Figure 1A**) and so the mean response for each animal was determined by averaging across these sessions. Data from 5 of the 24 ketamine exposure sessions (each animal received 3 ketamine exposure session) were eliminated from the analysis due to poor recording quality (1 or 2 sessions were dropped from 3 of the 8 rats). Inspection of these figures indicates strong gamma-band activity induced in M1 and HFO activity in dorsal and ventral striatum. In contrast, hippocampal responses appeared decidedly broadband for frequencies > 30 Hz. The time-course of the mean response (*n* = 8 rats) in each canonical frequency band is presented in **Figure 2B**.

**FIGURE 3:**
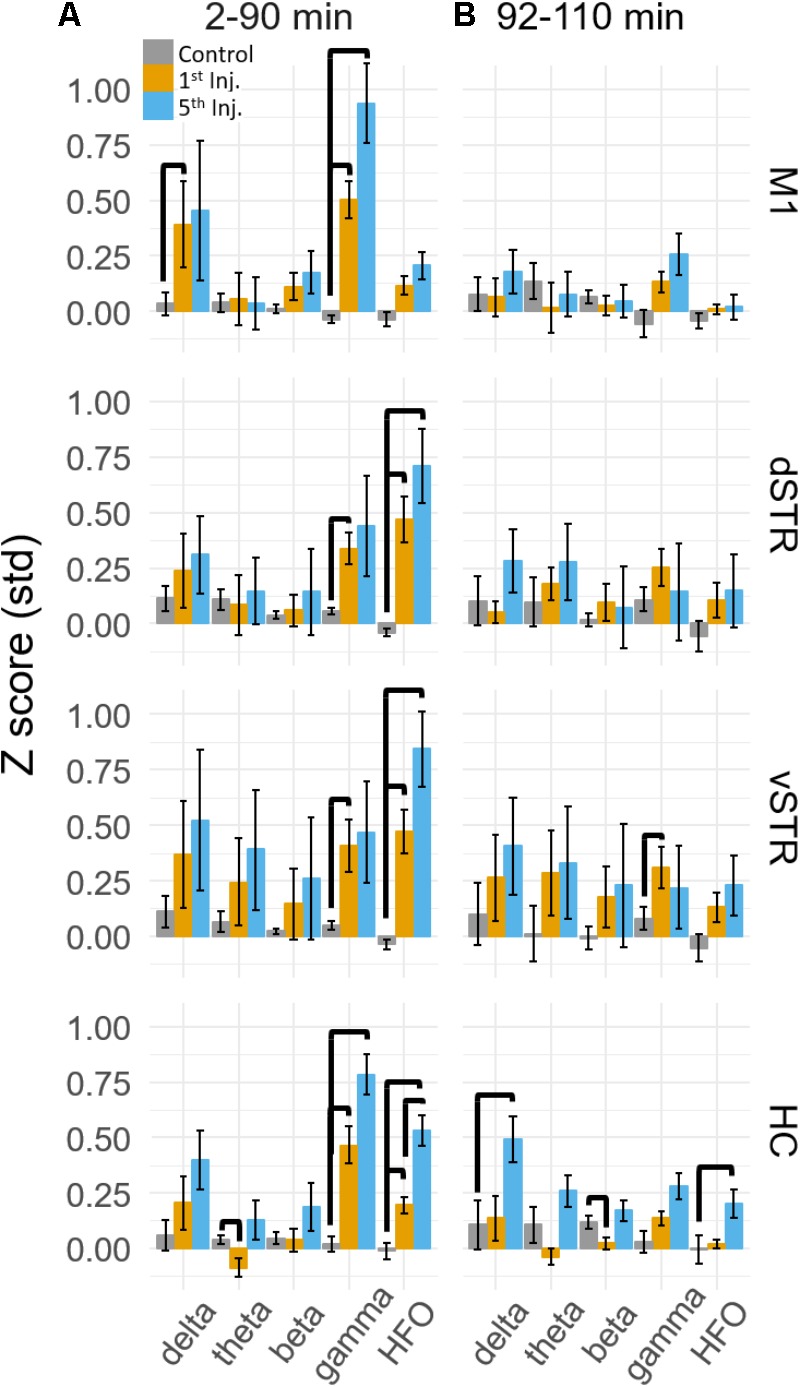
Ketamine-induced oscillatory activity. **(A)** Mean (±SEM) spectral power (*n* = 8 rats) following ketamine and saline injections during the 2–90 min post-injection period following the first (orange) and fifth (blue) injections. Bold bars indicate significant between-subject differences vs. controls and significant within-subject differences in either the 1st or 5th ketamine injections (paired *t*-test, Holm–Bonferroni correction, horizontal bars indicate significant effects at *p* < 0.05). **(B)** As in **(A)** but for the 92–110 min post-injection period. Significant increase in delta, beta, and HFO bands were observed in the hippocampus.

Spectral responses at canonical frequency bands were analyzed, and comparisons were made between the effects of ketamine and saline injection. Spectral power was measured in units of standard deviations (*Z* scores) above or below baseline spectral power (see Materials and Methods). Statistical comparisons for ketamine-triggered oscillatory power measured during control and ketamine sessions are summarized in **Figures 3A,B** for the 2–90 and 92–110 min post-injection intervals. These two intervals were chosen given previous work indicating that ketamine is metabolized ∼1 h post-injection (Páleníček et al., 2011). Thus, these intervals represent conservative estimates for periods when ketamine was (2–90 min) or was not (92–110 min) metabolically active. Black bold lines indicate significant comparisons between ketamine (orange and blue bars) and control saline (gray bars) sessions (*t*-test, *p <* 0.05, Holm-Bonferroni correction), and significant paired comparisons between ketamine injection 1 (orange) and injection 5 (blue, paired *t*-test, *p <* 0.05, Holm-Bonferroni correction).

A main effect of ketamine was observed during the 2–90 min post-injection interval in all brain regions [**Figure 3A**, Two-way ANOVA (drug, frequency), *p <* 0.05 following Holm–Bonferroni correction for 8 comparisons, *F* and *p*-values are in **Supplementary Table 1A**]. During the 92–110 min interval, however, a strong main effect of ketamine was only observed in the hippocampus [**Figure 3B**, *F*(1,55), *p* = 0.0006, η^2^ = 0.25], indicating that the lasting impact of extended exposure is in this brain region. *Post hoc* analysis also indicated that delta (*t*-test, *p* < 0.004, Holm–Bonferroni correction) and HFO power (*p* < 0.031) increased relative to the baseline period during the 92–110 min interval.

*Post hoc* comparisons revealed that during the 2–90 min interval, ketamine increased gamma power in M1 and HFO power in dSTR and vSTR relative to saline control (**Figure 3A**, *t*-tests, *p <* 0.05 following Holm–Bonferroni correction). Gamma power also increased in the dSTR and vSTR following the 1st and 5th injection, and delta power increased in M1 after the 1st but not the 5th injection. In the hippocampus, ketamine injections increased gamma and HFO power; however, inspecting the spectral responses in **Figure 2A** suggests that this reflects increased broadband power for most frequencies > 30 Hz. This differs from the discrete narrowband increases in HFO and gamma power observed within M1 and the striatum. Thus, ketamine-induced oscillatory activity in the hippocampus is “noisier” and less focal relative to corticostriatal HFOs and gamma oscillations and lasts longer (into the 92–110 min interval).

### Repeated Ketamine Injections Significantly Increased High-Frequency Oscillatory Power in the Hippocampus

A main objective of this study was to determine if extended exposure to ketamine produced a lasting change in oscillatory activity. This was investigated by determining if oscillatory power changed from the first to the fifth injection. As described in the previous section, ketamine increased gamma in M1, HFO’s in striatum, and broadband (>30 Hz) activity in the hippocampus, and these effects were observed during the 2–90 min interval following injections 1 and 5 (**Figure 3A**). Although the mean power in all of these bands appeared to increase from injection 1 to 5, the increase was only significant for the HFO band in the hippocampus (*post hoc* paired *t*-test, *p*_HFO_ = 0.007, Holm–Bonferroni correction). No significant differences between injections 1 and 5 were observed during the 92–110 min post-injection interval (**Figure 3B**), although, as described in the previous section, oscillatory power in the delta and HFO bands in the hippocampus were larger relative to pre-injection baseline.

### Prior History of Extended Exposure to Ketamine Does Not Alter Resting Oscillatory Activity

**FIGURE 4:**
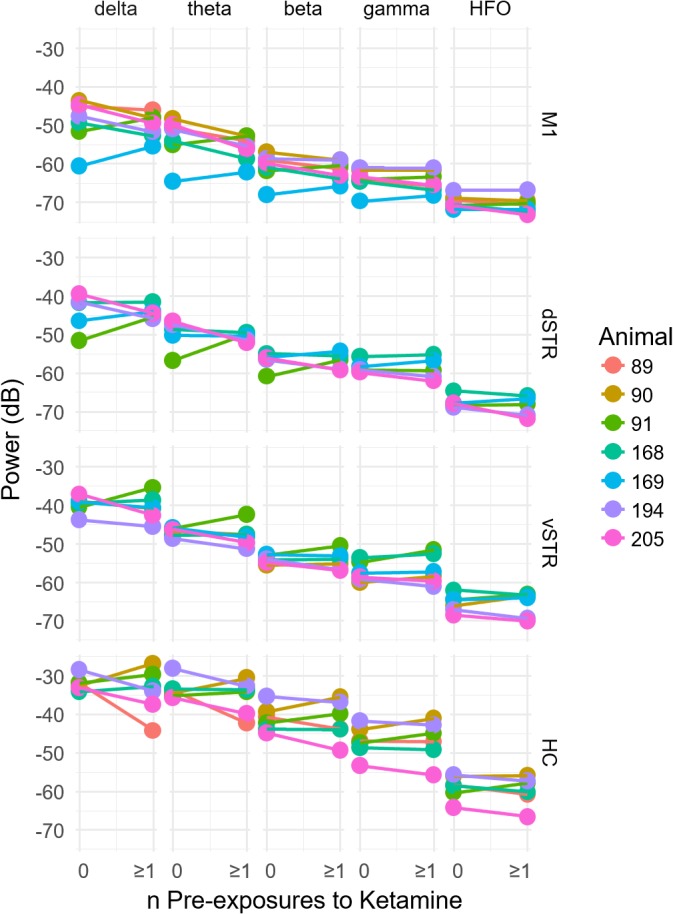
Prior history of extended exposure to ketamine does not alter resting oscillatory activity. The effect of the previous history of ketamine exposure on baseline oscillatory activity was investigated by comparing the oscillatory power during the –32 to –2 min interval preceding the first injection on each recording session. Sessions were separated by at least 1 week. The *x* axis of each plot indicates the number of previous ketamine exposure sessions (0 indicates days that the rat had no prior ketamine exposure session). A significant difference between the number of ketamine sessions received and oscillatory power (*y* axis) would suggest that prior ketamine exposures produces persistent changes in oscillatory activity that last at least 1 week, which was the time between ketamine sessions. Individual plots are organized by region (row) and frequency band (column). A within-subject analysis (within subject = number of ketamine sessions, between subject = animal) identified no significant relationships between the number of previous ketamine exposure sessions and oscillatory power at any frequency band (paired *t*-test, *p* > 0.05, *n* = 7).

Because exposure to ketamine can produce weeks-long improvement in symptoms of chronic pain and treatment-resistant depression, we explored the hypothesis that 10-h exposure to ketamine can produce measureable changes in oscillatory power that lasts for at least 1 week. This was assessed by comparing oscillatory power during the pre-injection period (-32 to -2 min prior to injection 1) for sessions in which rats had either no prior ketamine exposure sessions to sessions when the rats had at least one 10-h ketamine exposure (**Figure 4**). One animal was removed from the original *n* = 8 animals for this analysis because 2 of the 3 ketamine exposure sessions had poor recording quality. Consequently, it was not possible to investigate the effects of multiple ketamine exposures on oscillatory activity using this particular animal. Comparisons were made for each canonical frequency band (column) and brain region (row). A within-subject analysis (within subject = number of ketamine sessions, between subject = animal) identified no significant relationships between the number of previous ketamine exposure sessions and oscillatory power in any frequency band (paired *t-*test, *p* > 0.05, *n* = 7).

### Acute Ketamine Exposure Increases Locomotion, but This Increase Is Not Enhanced by Extended Exposure

Low-dose ketamine exposure increases locomotion and ataxic behaviors in rodents (Nicolás et al., 2011); however, motor activity is likely not causing the observed increase in HFO and gamma power as changes in locomotion lag behind ketamine-induced changes in HFO power (Caixeta et al., 2013). We also observed that ketamine increased locomotion relative to saline injection (*t*-test, *p* = 0.0015, *d* = 4.0, *n* = 8 rats), and this increase was manifest as an initial bout of ataxic behaviors (e.g., rearing and falling over) followed by increased locomotor activity and circling in the home cage.

**FIGURE 5:**
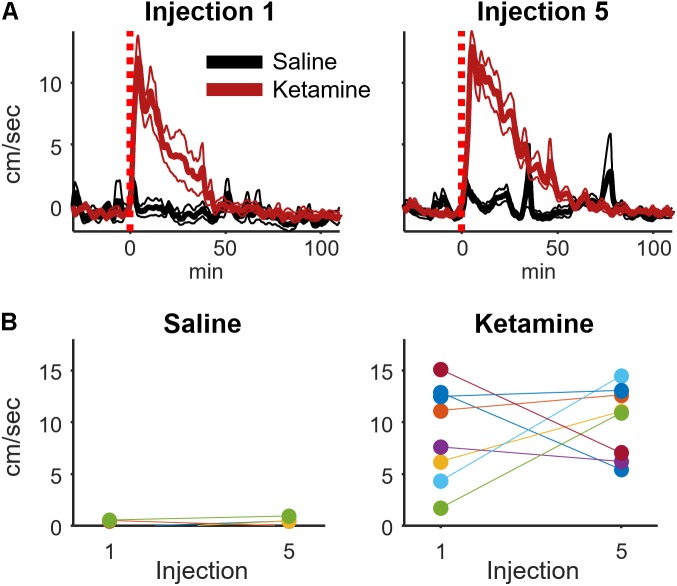
Ketamine-induced locomotion on the 1st and 5th injection. **(A)** Movement speed during each recording session was measured for periods surrounding ketamine or saline injection (bin size = 8 s, convolved with a 80 s Hanning window). This figure displays the mean and ±SEM across sessions (ketamine: *n* = 19, saline: *n* = 11 sessions from 8 rats) for the first and fifth injection. **(B)** To determine if movement speed was affected by multiple ketamine injections, average speed during the 2–20 min post-injection interval was analyzed and averaged for each rat. A strong effect of drug (ketamine vs. saline) was observed following injections 1 (*t*-test, *p* = 0.0015, *n* = 8 rats) and 5 (*p* = 0.00006, *n* = 8 rats). The effect of multiple injections was determined by comparing within-subject responses on the 1st and 5th injection using paired *t*-tests. Neither saline nor ketamine groups exhibited a significant difference in mean movement speed between injections 1 and 5 (paired *t*-test, saline: *p* = 0.50, *n* = 7 rats; ketamine: *p* = 0.59, *n* = 8 rats).

It was conceivable that the motor-activating effects of ketamine are enhanced by repeated ketamine exposures during an experimental session. Such increased locomotor activity could contribute to the rise in high-frequency broadband hippocampal power from injections 1 to 5 (**Figure 3**). To investigate this possibility, a within-subject analysis was performed that compared mean locomotion during the 2–20 min interval following injections 1 and 5 (**Figure 5**). Locomotion was quantified as the absolute value of the first derivative of the x and y position of the rat (cm/sec). This analysis indicated that ketamine-induced locomotion did not increase from injection 1 to 5 (**Figure 5B**, paired *t*-test *p* = 0.59, *n* = 8 animals).

### Ketamine-Induced Changes in Cross-Frequency Coupling

**FIGURE 6:**
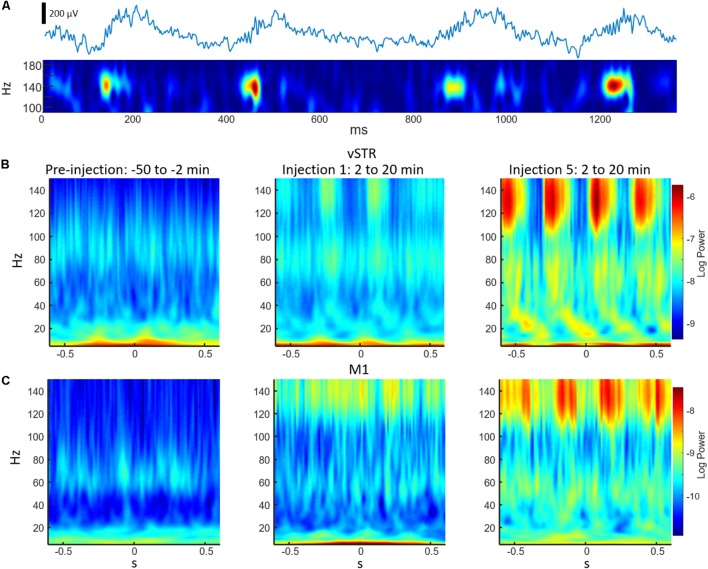
Examples of cross-frequency coupling following ketamine injections. **(A)** The top trace is an unfiltered 2 s representative local-field recording acquired from the vSTR during the 2–20 min post-injection interval following injection #5. The bottom trace is a wavelet spectrogram of the same signal. The spectrogram highlights the high-frequency (140 Hz) oscillations nested in a slower (∼3 Hz) oscillation. **(B)** Average wavelet spectrogram acquired from the vSTR aligned on the time of the troughs of the slower (1–4 Hz) oscillation for the pre-injection baseline period (first column) and for the 2–20 min intervals following the first and fifth injections. This example illustrates the capacity of ketamine to enhance CFC. **(C)** As in **B**, but for average wavelet spectrogram of the M1.

**FIGURE 7:**
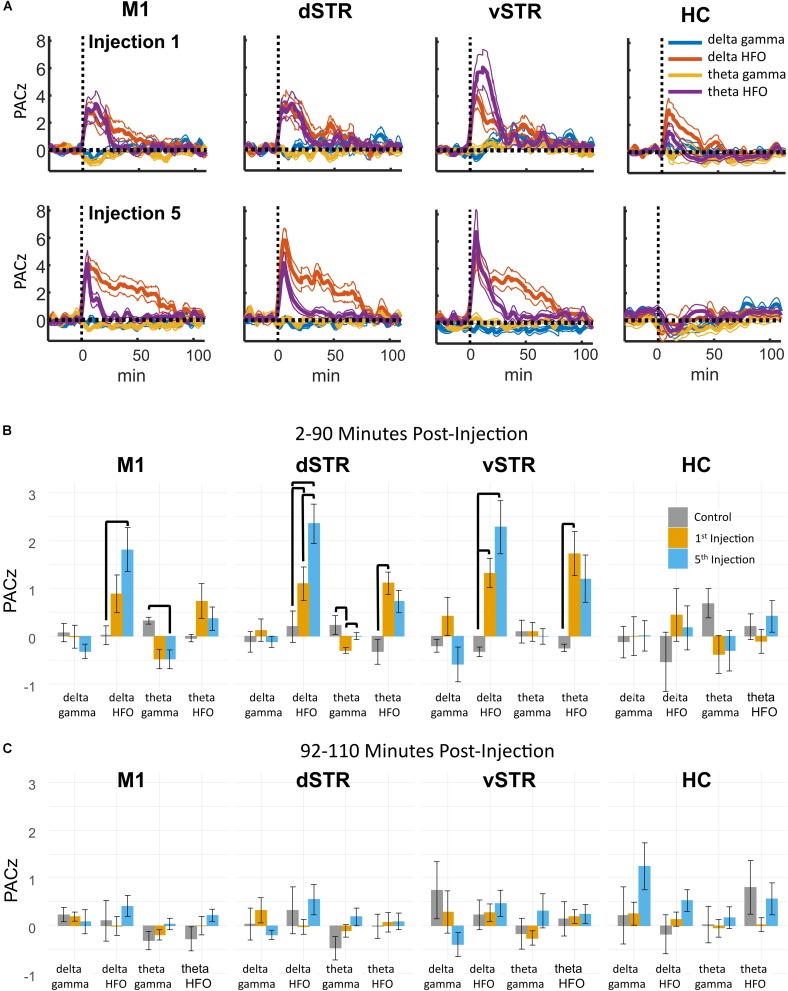
Ketamine-induced cross-frequency coupling. **(A)** The time course of CFC following the first (top row) and fifth (bottom row) ketamine injection (mean, ± SEM, *n* = 8 rats). Ketamine increased delta-HFO and theta-HFO CFC. Inspection of these responses indicated no delta-gamma and theta-gamma CFC despite strong ketamine-associated gamma oscillations in M1. The duration of delta-HFO CFC appeared to increase from injection 1 to injection 5 in M1, dSTR, and vSTR. **(B)** Comparison of CFC following injections 1 and 5. Mean and ± SEM CFC following ketamine and saline injections during the 2–90 min post-injection period following the first (orange) and fifth (blue) injections. Bold bars indicate significant between-subject differences vs. controls and significant within-subject differences in either the 1st or 5th ketamine injections (paired *t*-test, Holm–Bonferroni correction). Paired-comparison plots are provided in **Supplementary Figure 2**. **(C)** As in **B**, but for the 92–110 min post-injection period.

Phase-amplitude CFC measures the degree to which the phase of a low-frequency oscillation modulates the amplitude of a high-frequency oscillation. Such cross-band modulation may impact information processing and plasticity by organizing the timing of ensembles of neurons (Canolty and Knight, 2010; Lisman and Jensen, 2013). We investigated the effects of acute and extended low-dose ketamine exposure on within-region CFC. Representative examples of CFC measured during individual recording sessions are presented in **Figure 6**. The example in **Figure 6A** indicates strong ketamine-induced delta-HFO CFC in vSTR. **Figures 6B,C** present the average wavelet spectrogram aligned to the time of the trough of delta oscillations (1–4 Hz) in the vSTR and M1. These examples suggest a progressive increase in delta-HFO CFC from injection 1 to injection 5. The averaged time-course of ketamine-induced CFC following the first and fifth injection in all regions is presented in **Figure 7A**. Inspection of these responses indicates that ketamine induced a rapid increase in delta-HFO and theta-HFO CFC in corticostriatal regions, and these effects lasted from 20 to 90 min. Furthermore, the duration of ketamine-induced delta-HFO increased from injection 1 to injection 5, suggesting that prolonged exposure enhanced ketamine’s capacity to induce delta-HFO CFC. Ketamine did not induce CFC in the hippocampus, a result that is consistent with the observation that ketamine induced a non-specific wide-band signal on the hippocampal electrodes (**Figure 2**).

Statistical analyses of the effect of acute and prolonged exposure (from injection 1 to 5) on CFC are summarized in **Figure 7B**. Thick black lines indicate significant differences between saline (gray) and ketamine injection conditions or significant differences between injections 1 (orange) and 5 (blue). ANOVA (drug, frequency) was used to identify main effects and interactions, and result from the ANOVA are presented in **Supplementary Table 1B**. To summarize, main effects and interactions between drug and frequency were observed in M1, dSTR, and vSTR, but not the hippocampus. *Post hoc* tests indicated that sub-anesthetic ketamine produced a significant increase in delta-HFO CFC in dSTR and vSTR during the 2–90 min post-injection interval relative to saline (**Figure 7B**).

### Pronged Exposure to Ketamine Enhances Cross-Frequency Coupling in the Dorsal Striatum During the 2–90 min Post-injection Period

Paired comparisons between injections 1 and 5 were performed to determine if extended exposure to ketamine enhanced CFC. Paired comparisons were performed using a within-subject ANOVA (drug, frequency) followed by paired *t*-tests (see **Supplementary Table 1B** for all *F* and *p*-values). This analysis revealed that ketamine-induced delta-HFO CFC in dSTR increased from injection 1 to 5 (**Figure 7B**, *post hoc* paired *t*-tests *p* = 0.02, *d* = 1.4, Holm–Bonferroni corrected). In addition, theta-gamma CFC in dSTR decreased following the first injection (*p* = 0.003); however, this effect was not significant following the 5th injection (*p* = 1.0, Holm–Bonferroni correction). Paired-comparison plots for delta-HFO CFC for each region are provided in **Supplementary Figure 2**.

These data indicate that prolonged exposure enhances the capacity of ketamine to increase delta-HFO CFC in dSTR; however, analysis of responses during the 92–110 min post-injection interval did not identify any significant effects (**Figure 7C**). Consequently, the increase in delta-HFO CFC is likely mediated by ketamine’s direct action on corticostriatal receptor systems and not through ketamine’s metabolites or through entrained persistent activity.

### Ketamine-Induced Gamma in M1 and Locomotion Are Reduced by D1R Antagonist SCH-23390

**FIGURE 8:**
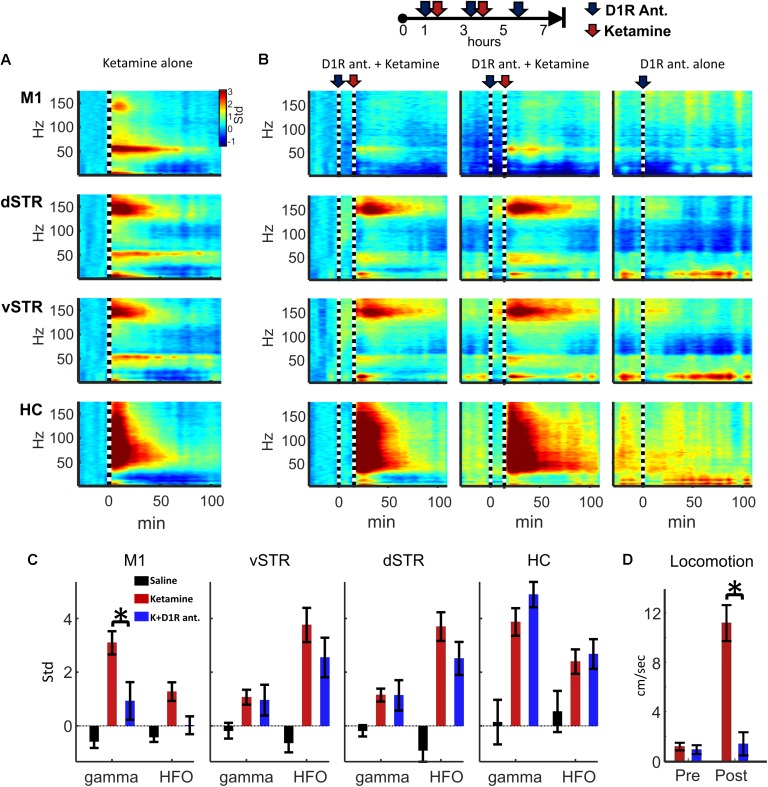
Ketamine-induced oscillations and movement following D1R antagonism. **(A)** Average power spectral responses to a single ketamine injection (20 mg/kg, i.p., *n* = 16 sessions and 6 rats). **(B)** Top: Schematic timeline of the injection procedure for pharmacological evaluation of the effects of D1R antagonist SCH-23390 (1.0 mg/kg). Bottom: The leftmost 2 columns indicate power spectral responses aligned to ketamine injection (20 mg/kg, vertical dashed line) administered 15 min after injection of SCH-23390. The last column indicates responses to D1R antagonist delivered alone (no ketamine injection). **(C)** Ketamine-induced gamma oscillations in M1 were reduced when D1Rs were blocked. Ketamine-evoked oscillatory power was measured during the 2–20 min post-injection period when ketamine was administered alone (red), after SCH-23390 (blue) or after saline injection. Comparisons between ketamine and ketamine + SCH-23390 conditions indicated that SCH-23390 significantly reduced ketamine-induced gamma and HFOs in M1 (*p* = 0.03, *n* = 6 rats, Student’s *t*-test with Holm–Bonferroni correction). **(D)** SCH-23390 eliminated ketamine-induced locomotion during the 2–20 min post-injection period (Student’s *t*-test, *p* = 0.0002). Error bars indicate SEM.

Experiment 2 investigated whether oscillatory or locomotor activity induced by ketamine was produced by activation of D1 receptors. To test this, ketamine (20 mg/kg, i.p.) was administered 15 min after the injection of D1R antagonist SCH-23390 (1.0 mg/kg, i.p.). The 15-min interval allowed the D1R antagonist to reach peak blocked of its targets. Spectral responses in **Figures 8A,B** show oscillatory responses to ketamine injection when ketamine was delivered either alone or after SCH-23390 injection. We tested the hypothesis that D1R antagonism would reduce ketamine-induced gamma or HFO activity (e.g., **Figure 2A**). Comparison of ketamine to the ketamine + SCH-23390 conditions indicated that SCH-23390 reduced ketamine-induced gamma in M1 (*p* = 0.03, *n* = 6, Student’s *t*-test with Holm–Bonferroni correction), but SCH-23390 did not alter gamma or HFOs in dSTR, vSTR, or HC (**Figure 8B**). Furthermore, administration of SCH-23390 eliminated ketamine-induced locomotion (**Figure 8D**, Student’s *t*-test, *p* = 0.0002). Thus, while M1 gamma produced by ketamine may involve activation of D1 receptors, ketamine’s capacity to induce HFOs and gamma in the striatum and hippocampus does not appear to be D1R mediated or the product of locomotion (**Figure 8D**).

## Discussion

Although sub-anesthetic infusions of ketamine are increasingly being used to treat chronic pain and treatment-resistant depression, the effects of prolonged exposure on neural synchrony are not understood. Using a spaced-injection procedure, we show that 10-h exposure enhances ketamine-induced broad-band gamma power in the hippocampus and increases ketamine-associated delta-HFO CFC in the dorsal striatum. These changes were strongest during the 2–90 min post-injection intervals that followed each of the five successive injections. In motor cortex and striatum, oscillatory activity was indistinguishable from baseline during the 92–110 min post-injection interval, suggesting that prolonged ketamine exposure potentiates acute responses to ketamine, but does not induce a novel or long-lasting pattern of synchrony that persists beyond ketamine’s period of metabolic action in these regions. Furthermore, and contrary to our original hypothesis, prolonged exposure did not increase the strength or extend the duration of ketamine-evoked HFOs in the striatum. In contrast, oscillatory responses in the hippocampus extended to the 92–110 min post-injection interval. This suggests that processes lasting longer than ketamine’s acute action on receptors, such as the activity of ketamine’s metabolites (Sleigh et al., 2014; Zanos et al., 2016), could have a stronger impact on hippocampal activity.

Moreover, these data indicate that ketamine’s acute effects on neuronal synchrony differ considerably in the cortex, striatum, and hippocampus. For example, where ketamine produced wide-band desynchronized activity in the hippocampus, responses in motor cortex and striatum were more narrow-band. Specifically, ketamine enhanced delta-HFO CFC, narrow-band gamma, and HFO power in motor cortex and striatum. It is therefore conceivable that ketamine exerts region-specific effects on oscillatory activity that impact the timing of action potentials in cortical, striatal, and limbic circuits involved in motivation and episodic memory formation.

### Ketamine-Induced Gamma in Motor Cortex

Injections of ketamine increased gamma power in M1, and this increase lasted for 40–60 min following each injection. Gamma oscillations may organize the timing of action potentials (van der Meer and Redish, 2009; Buzsáki and Wang, 2012), which can impact neural plasticity and information transmission between brain regions (Colgin et al., 2009). Although circuit-level mechanisms underlying cortical gamma generation are debated, it is generally believed that interactions between parvalbumin-(PV) expressing GABAergic neurons or interactions between PV neurons and principal cells are involved (Buzsáki and Wang, 2012). Although the mechanisms underlying ketamine-triggered gamma are not well understood, there is evidence that ketamine’s action on NMDARs plays a role in gamma generation as administration of selective NMDAR antagonists increase cortical gamma (Pinault, 2008), and ablation of NMDARs on PV neurons increases gamma (Korotkova et al., 2010). Inactivation of NMDARs by ketamine may preferentially reduce excitation of GABAergic neurons, resulting in the disinhibition of principal cells (Homayoun and Moghaddam, 2007) and consequently increase extracellular glutamate release from principal neurons (Moghaddam et al., 1997). Increased principal neuron activity and glutamate may increase gamma through the activation of metabotropic glutamate receptors (Whittington et al., 1995). Finally, dopamine D1Rs may be involved in gamma generation in M1 (Özkan et al., 2017). Ketamine-initiated gamma may also involve D1Rs as we observed that ketamine-induced locomotion and gamma in M1 were eliminated when ketamine was delivered after delivery of a D1R antagonist (**Figure 8**). This result further supports the involvement of deep-layer PV neurons in gamma generation as D1Rs are localized in layer VI (Weiner et al., 1991; Gaspar et al., 1995) and deep-layer PV interneurons are preferentially innervated by dopamine projections (Descarries et al., 1987; Gaspar et al., 1991; Williams and Goldman-Rakic, 1993; Vitrac et al., 2014). A role of D1Rs in ketamine-mediated gamma may be specific to M1 as D1R antagonism did not affect ketamine-induced oscillatory activity in NAc, an observation that is consistent with results from a previous investigation of the effects of D1R and D2R antagonists on ketamine-induced oscillatory activity in NAc (Matulewicz et al., 2010).

Ketamine’s capacity to enhance M1 gamma suggests that ketamine treatment could impact disorders affecting motor cortex. In this regard, there is evidence that ketamine reduces LID in Parkinson’s patients and in animal models of Parkinson’s disease (Bartlett et al., 2016; Sherman et al., 2016), with LID being associated with high-frequency cortical gamma (∼80 Hz) (Halje et al., 2012; Dupre et al., 2016; Swann et al., 2016). This is interesting as ketamine-induced gamma is about 30 Hz slower than the high gamma associated with LID. This large difference in frequencies suggests that in ketamine-induced and LID-associated gamma are produced by distinct circuits. Conceivably, these circuits could interfere with each other when simultaneously activated resulting in reduced LID. Future single-unit studies using animal models of LID could investigate whether cortical circuits generating low and high gamma are distinct and interfere with each other in LID. Patients with PTSD also experience increased high-gamma activity in resting-state networks (Dunkley et al., 2014), and PTSD patients can respond positively to ketamine treatment (Feder et al., 2014). Consequently, it is conceivable that interactions between distinct gamma circuits could mediate ketamine’s impact on these conditions.

### Cross-Frequency Coupling and HFOs in Corticostriatal Circuits

Cross-frequency coupling may facilitate memory encoding and retrieval, and the transfer of information between brain regions (Canolty and Knight, 2010). Aberrant CFC is also implicated schizophrenia (Allen et al., 2011) and Parkinson’s disease (Belić et al., 2016). Consistent with other reports, we observed that sub-anesthetic ketamine triggers HFOs in the striatum and delta-HFO and theta-HFO CFC in dSTR and vSTR (Cordon et al., 2015). Furthermore, prolonged exposure enhances the duration of delta-HFO CFC in dSTR (**Figure 7**). The mechanisms that produce striatal HFOs and delta-HFO coupling are not understood; however, the generator of HFOs may originate in the NAc as inactivation of the NAc with tetrodotoxin abolishes ketamine-induced HFOs (Olszewski et al., 2013). The locus and mechanism of striatal delta oscillations are less well understood. Delta is typically associated with slow-wave sleep and is thought to be generated by cortical and thalamic circuits (Amzica and Steriade, 1995; Lõrincz et al., 2015). Delta during sleep organizes the timing of thalamocortical sleep spindles (8–15 Hz) and hippocampal sharp-wave ripples (∼150 Hz), two oscillations that are associated with memory consolidation (Reviewed in Mölle and Born, 2011). In contrast to sleep-associated delta, the delta-HFO activity that was observed in the present study occurred during waking locomotion, indicating that this form of delta differs from slow-wave sleep-associated delta. Delta in waking rats has been observed in the ventral tegmental area (VTA) and the prefrontal cortex, and this delta activity emerged when rats performed a spatial working-memory task (Fujisawa and Buzsáki, 2011). Future studies involving inactivation of VTA or prefrontal cortex following ketamine administration could determine if ketamine-induced delta-HFO CFC is produced by the prefrontal cortex or the VTA.

### Ketamine Induces Broad-Band Gamma and Persistent Activity in the Hippocampus

The hippocampus plays a central role in the consolidation of episodic memory and in the processing of configurations of items in space and in time. Hippocampal dysfunction is also associated with major depression (Schmaal et al., 2016) and PTSD (Dunkley et al., 2014). Notably, these two disorders can be treated with low-dose ketamine infusion (Berman et al., 2000; Diamond et al., 2014; Feder et al., 2014). There is evidence that narrow band low- and high-gamma can differentially direct the flow of signals through intra-hippocampal pathways in order to support memory recall and encoding (Colgin et al., 2009). We observed that ketamine injections produced wide-band gamma (>30 Hz, **Figure 2**) during the 2–90 min post-injection interval. Such a response could interfere with the low- and high-gamma oscillations associated with memory storage and retrieval. This wide-band signal could also contribute to the observed reduction in CFC (**Figure 7**) in the hippocampus, a result that is in accord with predictions from computational modeling (Neymotin et al., 2011). The observed reduction in hippocampal CFC following ketamine injection is also consistent with experimental work where NMDAR ablation in CA1 reduces theta-gamma CFC in behaving mice (Korotkova et al., 2010).

The effects of acute low-dose ketamine exposure on neuronal synchrony suggest that ketamine exposure would reduce memory performance. Indeed, low-dose ketamine injections do impair spatial memory in an 8-arm maze during encoding and retrieval phases (Chrobak et al., 2008). Such impairment could result from the absence of a distinct gamma oscillation to organize the timing of action potentials. Reduced precision of action-potential timing along with ketamine-mediated NMDAR blockade could ultimately disrupt plasticity. It is interesting to consider that disorganized broad-band activity could have a beneficial effect by potentially “resetting” aberrant synaptic connectivity within the hippocampus that contributes to depression and PTSD.

Although our data did not identify CFC in the hippocampus, experimental work by Caixeta et al. (2013) indicates that moderate-to-high doses of ketamine (25–75 mg/kg) do increase hippocampal theta-HFO and theta-gamma CFC. Potential reasons for this difference include the larger ketamine doses used in the Caixeta study, and the use of high-density linear electrode arrays that allowed identification of layer-specific gamma generators. In contrast, our paired-electrode recordings measured signals from a larger hippocampal volume. Consequently, it is conceivable that the broad-band activity observed in our study reflects the integrated activity of multiple distinct hippocampal gamma generators. Future experiments using high-density electrode arrays would resolve this issue.

A final interesting feature of the hippocampal response to ketamine was that, unlike motor cortex and striatum, some ketamine-induced oscillations in the hippocampus (e.g., delta and HFO) persisted into the 92–110 min post-injection interval (**Figure 3B**). This suggests that ketamine’s effect on memory consolidation and spatial information processing may last longer than its effects on motor and reinforcement-driven behaviors.

### Ketamine’s Effect on Oscillatory Power and Cross-Frequency Coupling Was Restricted to the 2–90 min Post-injection Interval

Although low-dose ketamine significantly affected corticostriatal synchrony and CFC during the 2–90 min post-injection period, these effects were largely indistinguishable from baseline during the 92–110 min post-injection intervals (**Figures 3B**, **7C**). This was surprising as ketamine infusions in human patients can produce week-to-month long reduction in depressive symptoms in patients with treatment-resistant depression (Berman et al., 2000; Diamond et al., 2014). One explanation for the time-limited response is that ketamine initiates the gradual synaptic reorganization of circuits involved in the pathology (Phoumthipphavong et al., 2016). Such reorganization may be facilitated by ketamine-induced gamma synchrony as gamma can enhance neuronal plasticity by synchronizing the timing of action potentials in pre- and post-synaptic neurons (Hasenstaub et al., 2016). Synaptic reorganization may be further facilitated by ketamine’s capacity to increase BDNF production (Yang et al., 2013) and spine density in the medial frontal cortex (Phoumthipphavong et al., 2016).

## Conclusion

Our data demonstrate that 10-h ketamine exposure produces desynchronized broad-band gamma in the hippocampus and increases delta-HFO CFC in the dorsal striatum. Although single injections triggered strong HFOs and gamma in the striatum, these responses were not enhanced by prolonged ketamine exposure. The observation that prolonged exposure increased delta-HFO CFC in dorsal striatum indicates that extended exposure enhances coordination between striatal neurons and possibly facilitates spike-timing dependent plasticity and inter-regional communication. In contrast, we observed that ketamine-induced oscillations in the hippocampus were decidedly broadband, a result that suggests widespread desynchronization. Such broadband “noise” in the hippocampus could negatively affect coordination between neurons and reduce the strength of associative networks within the hippocampus. However, such disruptive effects could have positive implications if these associative networks contribute to pathologies such as depression and PTSD. An important next step is to determine if ketamine differentially alters coordination between neurons in cortical, striatal, and hippocampal circuits though single-unit ensemble recordings. Furthermore, combining ensemble recordings with animal models of PTSD, chronic pain, depression, and LID could identify circuit-level changes that underlie ketamine’s therapeutic effectiveness.

## Ethics Statement

This study was carried out in accordance with the recommendations of NIH guidelines for the Care and Use of Laboratory Animals, The Institutional Animal Care and Use Committee. This protocol was approved by The Institutional Animal Care and Use Committee.

## Author Contributions

TY manufactured electrode arrays, performed all surgical procedures, and collected all data. MB titrated and administered all substances to experimental animals. MS and SC performed analyses. TF and SS provided advisement to experimental protocol. All authors contributed to the review of this manuscript.

## Conflict of Interest Statement

TY, MB, MS, and SC declare that the research was conducted in the absence of any commercial or financial relationships that could be construed as a potential conflict of interest. SS and TF do have a pending patent application for the use of ketamine as a novel treatment for levodopa-induced dyskinesia associated with Parkinson’s disease.
