# Supplementary material for: Ten-Hour Exposure to Low-Dose Ketamine Enhances Corticostriatal Cross-Frequency Coupling and Hippocampal Broad-Band Gamma Oscillations
Source: Front Neural Circuits. 2018 Aug 13;12:61. doi: 10.3389/fncir.2018.00061 (PMC6099120; doi:10.3389/fncir.2018.00061)
Supplement: Supplementary file 1 [file Table_1.PDF]

A

Oscillatory Power

	2-90 minutes				92-110 minutes	
Region	Injection #	F Drug	p corrected	F Drug	p corrected
M1	1st	14.69	**0.0021**	0.094	1.0000
dSTR	1st	8.73	**0.0112**	2.75	0.5128
vSTR	1st	9.90	**0.0103**	6.087	0.1154
HC	1st	8.78	**0.0111**	0.15	1.0000
M1	5th	14.54	**0.0021**	2.51	0.5128
dSTR	5th	9.11	**0.0112**	1.81	0.5497
vSTR	5th	11.30	**0.0068**	4.58	0.2187
HC	5th	40.55	**0.0000**	18.52	**0.0006**

p corrected for 8 comparisons (Holm)

B

Cross-frequency Coupling

	2-90 minutes				92-110 minutes	
Region	Injection #	F Int	p int corrected	F Int	p corrected
M1	1st	4.57	**0.0264**	1.46	1.0000
dSTR	1st	5.42	**0.0192**	0.33	1.0000
vSTR	1st	5.11	**0.0206**	0.81	1.0000
HC	1st	3.23	0.0759	0.25	1.0000
M1	5th	10.80	**0.0003**	1.44	1.0000
dSTR	5th	9.10	**0.0011**	0.81	1.0000
vSTR	5th	8.37	**0.0017**	1.12	0.4589
HC	5th	1.46	0.2471	2.76	1.0000

p corrected for 8 comparisons (Holm)

Supplementary Table 1. (A) Results from ANOVA (drug, frequency) for main effect of drug following the first and fifth injection. Significant main effects are indicated as bolded p values. All p values are corrected for multiple comparisons using the Holm-Bonferroni correction (8 comparisons). No interactions were observed for oscillatory power and so F values are not shown. **(B)** In contrast, interactions were observed in CFC in most regions, most regions expressing no or only minimal main effects. Consequently, only F values for interactions are show. A small main effect of drug was observed in CFC for dSTR, injection 1 ($p = 0.045$), and vSTR, injection 5 ($p = 0.031$), but a stronger interaction effect was observed for these regions so the main effect was not evaluated.
