# Supplementary figures and images for: Ten-Hour Exposure to Low-Dose Ketamine Enhances Corticostriatal Cross-Frequency Coupling and Hippocampal Broad-Band Gamma Oscillations
Source: Front Neural Circuits. 2018 Aug 13;12:61. doi: 10.3389/fncir.2018.00061 (PMC6099120; doi:10.3389/fncir.2018.00061)

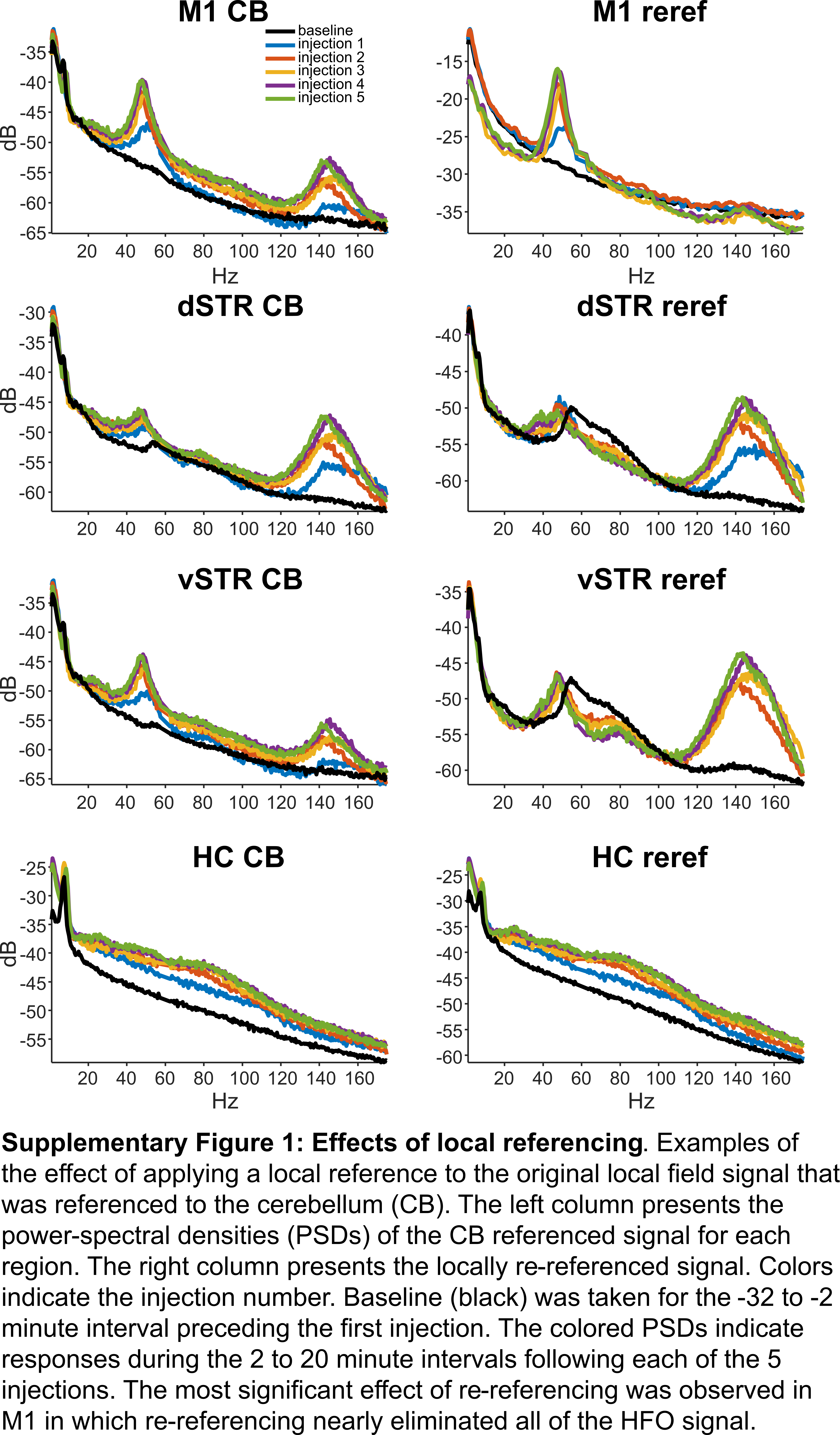

Supplement: Supplementary file 2 [file Image_1.tif]

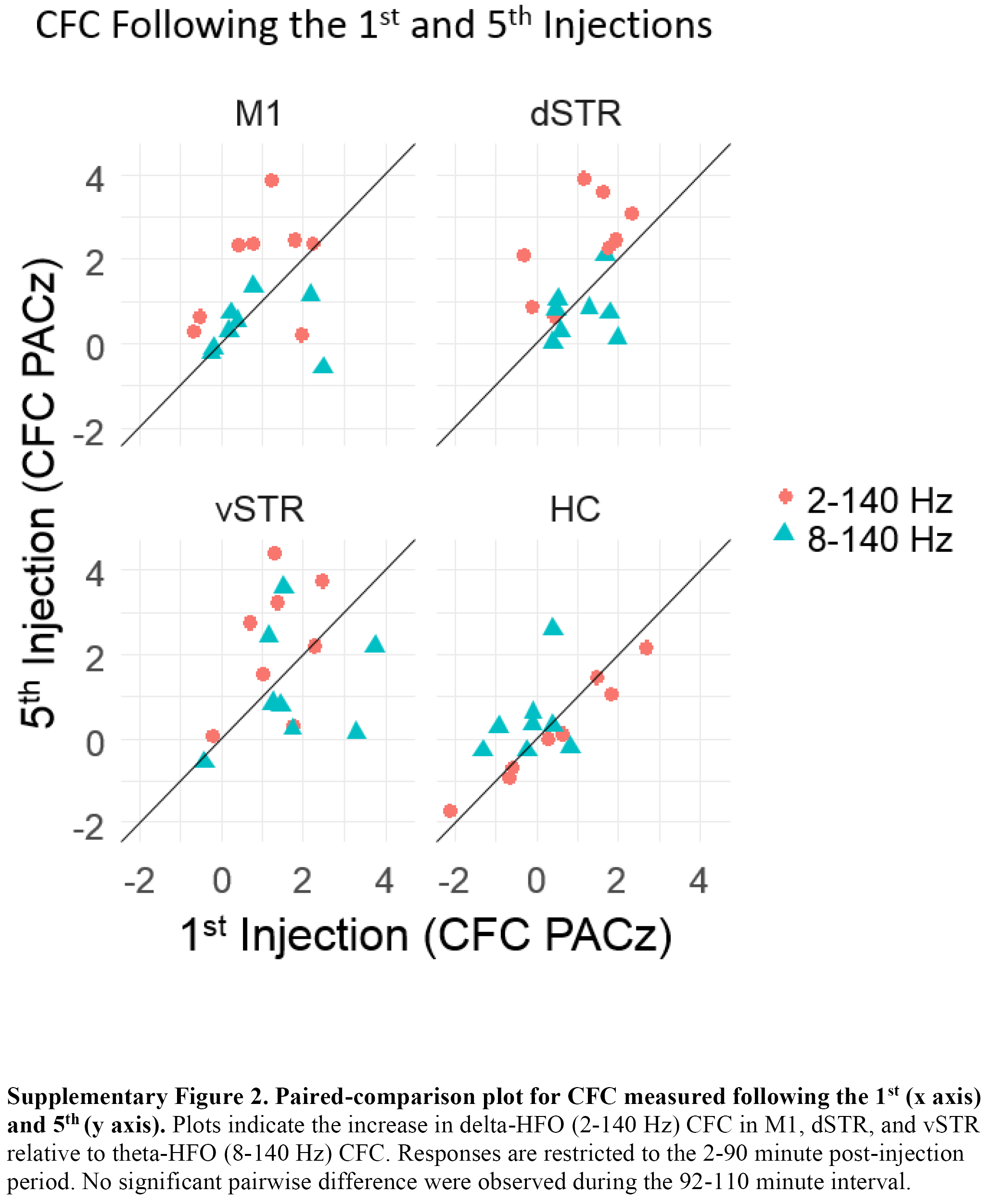

Supplement: Supplementary file 3 [file Image_2.TIF]
